# Neutralization Method of Ransomware Detection Technology Using Format Preserving Encryption

**DOI:** 10.3390/s23104728

**Published:** 2023-05-13

**Authors:** Jaehyuk Lee, Sun-Young Lee, Kangbin Yim, Kyungroul Lee

**Affiliations:** 1 Interdisciplinary Program of Information & Protection, Mokpo National University, Muan 58554, Republic of Korea; 2 Department of Information Security Engineering, Soonchunhyang University, Asan 31538, Republic of Korea; 3 Department of Information Security, Mokpo National University, Muan 58554, Republic of Korea

**Keywords:** entropy, format preserving encryption, neutralization, ransomware

## Abstract

Ransomware is one type of malware that involves restricting access to files by encrypting files stored on the victim’s system and demanding money in return for file recovery. Although various ransomware detection technologies have been introduced, existing ransomware detection technologies have certain limitations and problems that affect their detection ability. Therefore, there is a need for new detection technologies that can overcome the problems of existing detection methods and minimize the damage from ransomware. A technology that can be used to detect files infected by ransomware and by measuring the entropy of files has been proposed. However, from an attacker’s point of view, neutralization technology can bypass detection through neutralization using entropy. A representative neutralization method is one that involves decreasing the entropy of encrypted files by using an encoding technology such as base64. This technology also makes it possible to detect files that are infected by ransomware by measuring entropy after decoding the encoded files, which, in turn, means the failure of the ransomware detection-neutralization technology. Therefore, this paper derives three requirements for a more sophisticated ransomware detection-neutralization method from the perspective of an attacker for it to have novelty. These requirements are (1) it must not be decoded; (2) it must support encryption using secret information; and (3) the entropy of the generated ciphertext must be similar to that of plaintext. The proposed neutralization method satisfies these requirements, supports encryption without decoding, and applies format-preserving encryption that can adjust the input and output lengths. To overcome the limitations of neutralization technology using the encoding algorithm, we utilized format-preserving encryption, which could allow the attacker to manipulate the entropy of the ciphertext as desired by changing the expression range of numbers and controlling the input and output lengths in a very free manner. To apply format-preserving encryption, Byte Split, BinaryToASCII, and Radix Conversion methods were evaluated, and an optimal neutralization method was derived based on the experimental results of these three methods. As a result of the comparative analysis of the neutralization performance with existing studies, when the entropy threshold value was 0.5 in the Radix Conversion method, which was the optimal neutralization method derived from the proposed study, the neutralization accuracy was improved by 96% based on the PPTX file format. The results of this study provide clues for future studies to derive a plan to counter the technology that can neutralize ransomware detection technology.

## 1. Introduction

Ransomware is one of the malicious codes that restrict access to files by encrypting the files stored in the victim’s system and then requesting rewards from the victim for file recovery [1,2]. According to “The State of Ransomware 2021,” the 2021 annual ransomware survey conducted by the British security software company Sophos, 37% of all respondents in 30 countries, including the U.S., U.K., India, France, Germany, and Japan, responded that they suffered damage from ransomware in 2020. The global average amount they paid ransomware attackers was about $170,000. Nevertheless, even with a ransom payment for data recovery, only 65% of the encrypted data was decrypted. Moreover, the cost of recovering damage from the attack, including the amount paid to the attacker, amounted to a global average of $1.85 million. As such, when infected with ransomware, the ransom must be paid in exchange for data decryption. In addition, even if the ransom is actually paid, data decryption cannot be guaranteed [3].

Due to such damage, ransomware detection technology is essential. Many technologies have been studied to prevent infection and minimize damage. These ransomware detection technologies are classified into technologies that can prevent infection from ransomware and technologies that can detect files infected with ransomware in the system. Detection technologies for infection prevention include file-based detection [4], which identifies malicious signatures that appear in a specific file format; system-based detection that blocks malicious behavior and integrity verification [5]; resource-based behavior detection [6], which detects based on CPU and I/O throughput, and connection-based behavior detection [7], which detects ransomware by receiving the encryption key from the network. In addition, for the purpose of minimizing damage, entropy measurement-based detection methods that can detect files infected with ransomware in the system have emerged [4,5,6,7]. Despite these efforts, detection technologies for the prevention of ransomware infection have limitations in that it is difficult to detect new ransomware and variants. In addition, they have a high false positive rate. Moreover, the environment for detection is restricted. Furthermore, if entropy is low or manipulation is possible, there is a problem if the detection rate is low because the method of detecting files infected with ransomware in the system determines whether files are infected with ransomware based on the entropy of the file.

In order to overcome these problems of existing detection technologies as described above, sufficient analysis of existing ransomware detection technologies is required to minimize damage and prevent infection from ransomware. It is also necessary to consider the position of the attacker who creates the ransomware to come up with countermeasures. Based on this reason, recently, technologies that can neutralize entropy measurement-based ransomware detection methods have been studied [8,9]. A representative technique is a study that applies various encoding methods to each file format in order to lower the entropy of an encrypted file and derive an encoding method with optimal performance so as not to be detected as a file infected with ransomware.

Nevertheless, if the entropy of decoded encrypted files is measured after decoding, there is a drawback in that the encoded file encrypted by the ransomware can be detected because this neutralization technique can decode the encrypted file. Therefore, in this paper, research on detecting files infected with ransomware in the system was mainly analyzed based on the entropy of the file, and a more sophisticated method of neutralizing the ransomware detection technology was proposed from the attacker’s point of view. The proposed neutralization method can neutralize a ransomware detection method by freely modifying the entropy of the ciphertext as desired by the attacker in order to overcome the drawbacks of the encoding technique. Moreover, we proposed a method to neutralize ransomware utilizing the characteristics of format-preserving encryption, which has the advantage that there is no problem, such as the exposure of the neutralization method due to decoding, because the proposed method is possible without decoding.

The contributions of this study are as follows:

- To prevent infection from ransomware and minimize damage, existing ransomware detection methods were intensively analyzed, and the attacker’s position was considered to derive effective countermeasures. Through this, we propose a method to neutralize a more sophisticated ransomware detection method. This study is expected to provide results that, combined with previous studies, could help develop a method in the future that is used to counteract the technology that neutralizes ransomware detection technology.
- By intensively analyzing existing ransomware detection-neutralization methods, the weaknesses of previous studies were derived, and three neutralization methods to overcome those weaknesses were proposed.
- To propose a method of neutralizing the entropy measurement-based ransomware detection method, we analyzed the applicability of format-preserving encryption that could overcome the difficulties of applying cryptography algorithms in general and conform to the demands of the attacker. Finally, we proposed a sophisticated neutralization method.

## 2. Prior Knowledge and Related Works

This section describes the analysis results of ransomware detection-neutralization methods proposed in previous studies and introduces the prior knowledge and related research that are required to understand the proposed neutralization method. In prior knowledge, information entropy, which is the basic concept of the file encryption measurement-based ransomware detection method, and format-preserving encryption applied to the neutralization method proposed in this paper are explained. Moreover, related research describing a method to neutralize ransomware detection technology using encoding algorithms that can neutralize the file entropy measurement-based ransomware detection technology is described.

### 2.1. Prior Knowledge

Here, information entropy, ciphertext characteristics, and the relationship between entropy and the expression range of numbers are described as prior knowledge of the background of the proposed method.

#### 2.1.1. Information Entropy

Entropy, introduced by Shannon, is a term that expresses a degree of unpredictability or amount of uncertainty [10,11]. In information security or computer engineering, it is expressed as information entropy, meaning the expected amount of information. Information entropy means uniformity of data. If data are uniform, then entropy is high. Conversely, if data are not uniform, then entropy is low. This entropy has a value from a minimum of 0 to a maximum of 8 based on 8 bits.

#### 2.1.2. Ciphertext Characteristics

Here, the characteristics of the ciphertext used by ransomware producers are described. Encryption means converting plaintext into ciphertext using a cryptographic algorithm so that no one except for the person who has information on the key, which is secret information, can acquire the information needed to provide confidentiality [12,13,14]. Conversely, decryption is the reverse of encryption. Only a person who has information about the key can convert the ciphertext into plaintext. As such, the main purpose of cryptography is that only a person with a key can generate and extract ciphertext and plaintext. In addition, the cryptography algorithm is designed so that all values are distributed with the same probability without frequently appearing or biasing specific values to prevent a person who does not have a key from decrypting the ciphertext or guessing the plaintext. Namely, an excellent cryptography algorithm should have all data uniformly distributed.

After all, the ciphertext generated by the cryptographic algorithm has a high entropy. This means that if the ransomware producer creates an encrypted file, the entropy of the file is increased. Based on the characteristics of these ciphertexts, a number of methods have been studied to detect files infected with ransomware in the system by measuring the entropy of files [8,15,16].

#### 2.1.3. Correlation between Entropy and Number Expression Range

This paper proposes a new neutralization method to detect files infected with ransomware by measuring the entropy of files. When an existing detection method measures the entropy of files, if the measured entropy is similar to the entropy of the ciphertext, it is detected as a file infected with ransomware [15]. To neutralize this ransomware detection method, even if specific files are infected with ransomware and encrypted, it is possible to neutralize them by lowering the entropy generated based on the information entropy and ciphertext characteristics or by manipulating them to have a similar entropy of plaintext as described above in Section 2.1.1 and Section 2.1.2.

Based on these characteristics, we propose a new neutralization method that can manipulate entropy by transforming the expression range and form of numbers. Since the entropy of a file is generally measured based on 8 bits, it can be expressed from 0 to 255. For example, if one creates a file with the same distribution of all numbers from 0 to 255, the entropy is measured as eight because the data are uniform. This characteristic is a correlation according to the expression range of entropy and number. In this paper, the proposed method can manipulate the entropy to neutralize entropy-based ransomware detection methods by adjusting the expression range and form of numbers.

#### 2.1.4. Format-Preserving Encryption

Format-preserving encryption was proposed by Brightwell in 1997. Unlike existing cryptography algorithms, this algorithm has the same length and format as plaintext and ciphertext. Explaining the background of the appearance of format-preserving encryption, as the number of systems that collect and utilize personal information increases with the development of information and communication technology, the importance of encrypting personal information has also increased [17]. Nevertheless, in the case of encrypting personal information using a traditional block cipher algorithm, there is a problem in that the storage space is a waste because the length and format of data can be determined by the size of the block.

For example, credit card numbers, account numbers, and social security numbers (SSNs), which are representative of personal information, most often have a predetermined length and format. They generally consist of numbers within 10 digits. As a method to solve this problem, an encryption algorithm that does not depend on the size of a block is required to efficiently use data storage space. A format-preserving encryption that satisfies these requirements has emerged. The characteristic of format-preserving encryption is that the length and format of the ciphertext are not determined according to the size of the block as in the general block cipher algorithm, although the length and format of the plaintext and the ciphertext are the same. Due to this characteristic, if the plaintext is a letter or a number, the ciphertext can also be output as a letter or a number, which is effectively utilized in an environment or application where the length and form of data do not change, such as personal information.

Recently, the standardization of format-preserving encryption has been studied [18]. Format-preserving encryption such as FF1 and FF3-1 established as standards used terms of domain, radix, and tweak to maintain their format and length. The domain is a set of codes used for data. Radix is the number of domain codes. Tweak refers to information that is additionally input for the purpose of providing confidentiality. For binary, octal, and decimal data, radix and domain can be expressed as follows [19].

(1) radix=2, domain=0, 1

(2) radix=8, domain=0, 1, 2, 3, 4, 5, 6, 7

(3) radix=10, domain=0, 1, 2, 3, 4, 5, 6, 7, 8, 9

According to the above Formulas (1)–(3), the length and format of the ciphertext can vary depending on the radix of the format-preserving encryption. This feature has the result that an attacker generating the ciphertext can determine the length and format of the ciphertext as desired. In other words, the ransomware producer can arbitrarily determine the expression range of a number by changing the radix using format-preserving encryption. Through this, it is possible to manipulate the entropy of the ciphertext as desired. The core idea of this paper is that it is possible to neutralize the entropy measurement-based ransomware detection method based on characteristics of format-preserving encryption. Therefore, in this paper, in order to take advantage of the features and advantages of format-preserving encryption, FF1, standard format-preserving encryption, was chosen and used in the method to neutralize ransomware detection technologies.

### 2.2. Related Works

Existing ransomware detection technologies can prevent ransomware infection using various methods, including file-based detection methods, system-based detection methods, resource-based behavior detection methods, and connection-based behavior detection methods. However, these methods are limited in that they cannot effectively detect ransomware due to various problems. To solve this problem, recent studies have examined methods for identifying whether a file is encrypted after a system has been infected with ransomware rather than preventing ransomware infection.

As a related work, [20] proposed a zone division-based ransomware detection method to detect ransomware by separating areas containing file metadata, such as file headers, footers, signature information, and file contents that define file extensions in binary data. In [15], features for entropy distribution were derived for various file formats and machine learning models, such as KNN (K-Nearest Neighbor), linear regression, ridge regression, logistics regression, decision tree, random forest, SVM (Support Vector Machine), and MLP (Multi-Layer Perception), which were applied. These studies demonstrate that it is possible to detect ransomware-infected files with high accuracy. Finally, in [21], entropy calculation methods with optimal performance for identifying ransomware-infected files were derived by comparing various entropy calculation methods to identify files infected with ransomware. All of these studies utilized entropy to detect files infected with ransomware. However, if entropy could be manipulated, this would neutralize such detection techniques.

A representative detection method is an entropy measurement-based ransomware detection method that utilizes a characteristic where the entropy of an encrypted file is increased compared to a plaintext file when the file is encrypted. However, this method also has a problem in that the detection technology is neutralized if the ransomware producer manipulates the encrypted file to reduce entropy in any way. As a representative neutralization method using these problems, the method of neutralizing ransomware detection using encoding from the attacker’s point of view, as described in a prior study, was used in this study. In the prior study [8], base64 encoding was applied to lower the entropy of the encrypted file. To improve the neutralization performance of the prior study, various encoding methods, such as base32, base64, ASCII85, and URL encoding were applied to various file formats.

The core of these studies was to measure the entropy of plaintext, ciphertext, and encoded ciphertext. It has been verified that the entropy-based ransomware detection technology could be effectively neutralized by showing that the entropy of the encoded ciphertext is similar to plaintext [8,9]. Moreover, studies applying various encoding methods have verified that entropy differs according to the encoding method used to effectively neutralize the detection method compared to simply applying base64 encoding by deriving an optimal encoding method for each file format. The optimal method derived in a prior study [8] set the entropy threshold to 1.0 and showed an 84% improvement in its performance compared to a previous study in the CSV file format [8].

## 3. Limitation Analysis of Previous Studies and Experimental Configuration

This section points out the limitations of the previous study [8]. The encoding-based ransomware detection-neutralization method, the proposed neutralization method, and the experimental results were described to overcome these limitations. In terms of the experiment, the experimental environment, dataset configuration, and experimental goal of this study are presented here.

### 3.1. Limitation Analysis of Previous Studies

This section intensively analyzes the encoding-based neutralization method, which can neutralize the ransomware detection technology from the attacker’s point of view, as described in a previous study [8]. This study then draws the limitations of the encoding-based neutralization method. As described in Section 2.2. Related Works, the previous study applied a total of four encoding methods, including base64, to various file formats in order to neutralize the ransomware detection technology by lowering the entropy of the encrypted file. Since each encoding method has a different method of encoding data, the number of data that can be expressed varies. As a result, even with the same data, entropy is different for each encoding method. Based on these characteristics, an optimal encoding method with an entropy value most similar to that of plaintext was derived from four encoding methods. That is, if the optimal encoding method is applied, even if the entropy of the encrypted file is measured after being infected with ransomware, the entropy of the encoded file is almost similar to that of plaintext with a clear difference from the entropy of the ciphertext because the encoding algorithm can be applied to the encrypted file. This makes it difficult to detect files that are encrypted by ransomware. Despite these advantages, neutralization methods using encoding have the following obvious limitations.

Limitations: Aside from the text file format (.txt), most file formats have entropy differences. In this context, the entropy for each file format can be generalized because each file format includes a specific structure or additional processes such as compression. Based on the generalized entropy for each file format, from a defender’s point of view, the ransomware detection method can identify files with a significantly lower or higher entropy compared to the entropy of plaintext files infected files from ransomware. However, if encoding is applied, this makes it difficult to detect infected files from ransomware because the entropy of the plaintext files is similar to that of encoded files. Nevertheless, unlike the encryption algorithm, since the encoding algorithm can decode even without the key, which is secret information if the entropy of the encrypted file is measured after decoding, the infected file can be detected from ransomware.

**Figure 1:**
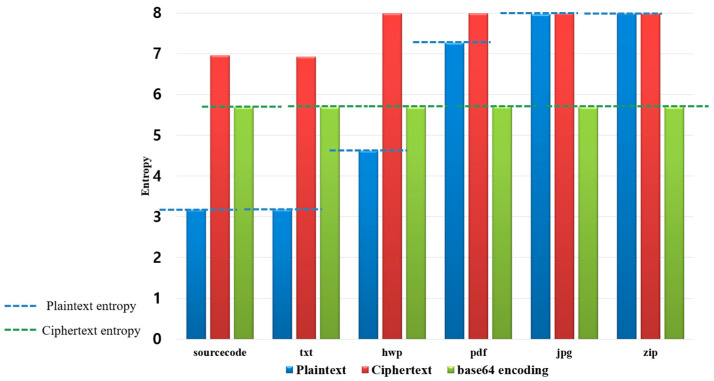
Limitations of previous studies (entropy difference between plaintext and base64 encoding).

Figure 1 shows an entropy plaintext, ciphertext, and after the application of base64 encoding for various file formats to intuitively show the limitations of the neutralization method using the encoding method.

Figure 1 shows the entropy plaintext, ciphertext, and after the application of base64 encoding for various file formats, such as the source code, txt, hwp, pdf, jpg, and zip file formats. For each file format, blue is the entropy of the plaintext, and purple is the entropy after applying base64 encoding, which was infected with ransomware to neutralize ransomware detection.

In particular, there is a large difference in entropy between the plaintext and base64 encoding for each file format. As mentioned in the above limitations, the neutralization method using encoding is the baseline for determining that the encoding for neutralization has been applied to the file with a significantly lower or higher entropy compared to the entropy of the plaintext file from the defender’s point of view. The figure intuitively shows these limitations.

**Figure 2:**
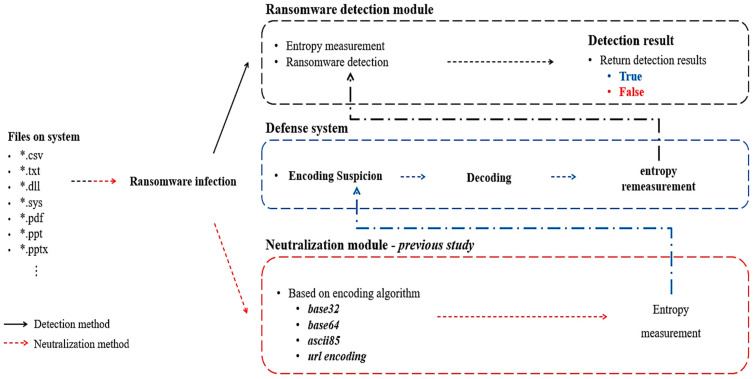
Detection procedure for encoded files encrypted by attackers according to limitations of previous studies.

Based on the above limitations, Figure 2 shows the process of detecting infected files with ransomware from a defender’s point of view.

Ransomware encrypts files of various file formats stored in the system and encodes encrypted files using the optimal encoding algorithm for each file format so that it is not detected by the defense system for detecting files infected with ransomware. On the other hand, the defense system determines whether the file is encoded. If it is an encoded file, it determines that detection is neutralized and decodes the encoded file. After decoding, the entropy of the encrypted file is measured to detect the file infected with the ransomware. The file infected with the ransomware is then detected by comparing the measured entropy with a threshold value for each file format based on the measured entropy of the encrypted file. Therefore, if the defense system identifies that the file is encoded, it can effectively detect the file infected with ransomware even if the encoding method described in a previous study [8] is applied. This is a limitation of previous studies.

### 3.2. Dataset Configuration and Experiment Goals

Here, in order to verify the method proposed in this paper, the configuration of the dataset used in the experiment and three experimental goals are described.

#### 3.2.1. Dataset Configuration

In this paper, we used the same dataset as in the previous study [8] to overcome the limitations of previous studies and to compare and analyze with the optimal neutralization method. The dataset used in the experiment was the GovDocs1 dataset, which was provided for forensic research. It had various file formats, including csv, doc, docx, dump, jpg, log, ppt, pptx, rtf, swf, txt, xls, xlsx, and zip file formats. Table 1 shows the types and numbers of file formats of the dataset used in the actual experiment.

#### 3.2.2. Experimental Goals

The proposed method had a total of three experimental goals. Based on the dataset, the results of previous studies were compared and analyzed through experiments. 

The first goal was to propose three neutralization methods to overcome the limitations of prior studies and derive a new neutralization method with optimal efficiency. The second goal was to compare and analyze the experimental results of the three proposed methods with those of previous studies. The third goal was to derive an optimal neutralization method of the entropy measurement-based ransomware detection technology based on the comparison and analysis results.

## 4. Proposed Neutralization Method

In this section, based on the experimental configuration and the comparison results with previous studies, as detailed in Section 3, we propose a neutralization method against ransomware detection methods and derive an optimal neutralization method.

### 4.1. Neutralization Methodology Using Format-Preserving Encryption

To overcome the problem that occurs after decoding, which is the problem of the neutralization method using the encoding algorithm, which has been described in a previous study [8], we proposed a new neutralization method using format-preserving encryption. In common symmetric key encryption algorithms, there is a clear difference between the entropy of the ciphertext and the entropy of the plaintext. 

Because of this, the detection system can detect whether the system has been infected by ransomware by measuring the entropy of the file. To overcome this limitation, a more sophisticated neutralization method should meet the following requirements: (1) it must not be decoded; (2) it must support encryption using secret information; and (3) the entropy of the generated ciphertext must be similar to that of plaintext.

**Figure 3:**
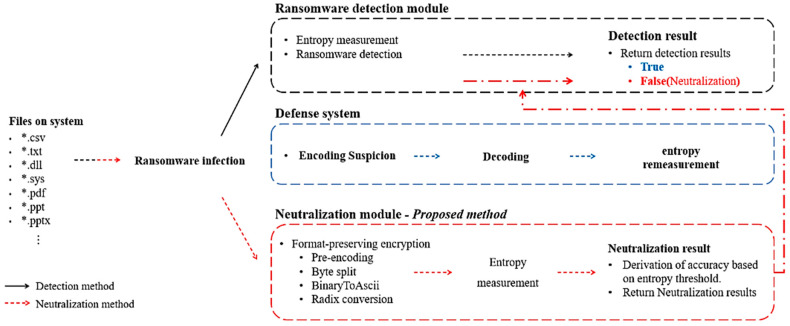
Methodology for neutralizing ransomware detection using format-preserving encryption.

An encryption algorithm that satisfies all these three requirements is format-preserving encryption. Since format-preserving encryption is an encryption algorithm, the proposed method is not decoded because it generates a ciphertext based on the key. Since the proposed method is not decoded, it satisfies requirements 1 and 2. Moreover, format-preserving encryption preserves the format of input and output by inputting decimal, hexadecimal, and characters and outputting the same format. This means that, in the end, the range of input data can be specified, and the entropy of the generated ciphertext can be adjusted using format-preserving encryption. Therefore, the proposed method satisfies requirement 3. Based on the analysis results, to neutralize ransomware detection technology, we determined in this paper that format-preserving encryption was the most suitable. Three methods were proposed for entropy manipulation. Figure 3 shows the methodology of the proposed ransomware detection-neutralization method.

If we analyze the neutralization reasonability, the neutralization method using the format-preserving encryption that we proposed could not apply the encoding algorithm. Thus, a decoding process was not required. Therefore, the ransomware detection system must measure entropy to detect files infected with ransomware. However, since encrypted files are almost similar to the entropy of plaintext, it is impossible to detect files infected by ransomware based on the entropy threshold. For these reasons, the neutralization method using format-preserving encryption is considered reasonable. To generate ciphertext with entropy almost to the entropy of plaintext and to specify the range of representable numbers in the input and output, we derived three techniques. These three derived techniques are shown in Table 2.

The three proposed techniques are Byte Split, BinaryToASCII, and Radix Conversion. All three techniques use format-preserving encryption as cryptographic algorithms. The first method, Byte Split, is a method that can lower the entropy of the ciphertext by splitting the bytes of the generated ciphertext. The second method, BinaryToASCII, is similar to the Byte Split method. It manipulates binary data to reduce the entropy of the ciphertext by replacing it with ASCII. The last method, Radix Conversion, actively utilizes the advantage of format-preserving encryption, which can control the expression range of numbers. It manipulates the radix to reduce the entropy of the ciphertext.

#### 4.1.1. Byte Split

The Byte Split technique splits bytes in order to reduce the entropy of the ciphertext and appear similar to the entropy of the plaintext. In order to reduce entropy, this technique basically follows the correlation of entropy according to the range of expressions of a number. Entropy is lowered by reducing the expression range of the number, which can be expressed by the existing one byte to the highest maximum possible. The range of representation for a number that can be expressed in one byte is from 0 to 255. If a ciphertext can be generated based on this range, ideally, the entropy can have a value close to eight. Therefore, this technique can reduce entropy by splitting bytes to adjust the range of representable numbers.

**Figure 4:**
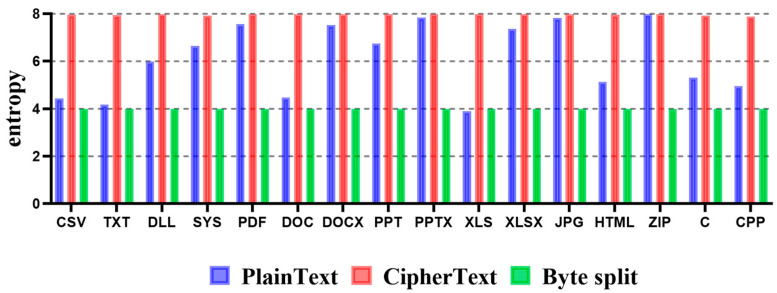
Entropy measurement results according to each file format using the Byte Split technique.

In detail, 8 bits, which are one byte, are separated by 4 bits, which are half bytes. Zero is inserted into the upper 4 bits of the first 4 bits separated. For example, if the value of one byte is 0x42, then 0x4 and 0x2 are separated into one byte each, and 0 is inserted into the upper 4 bits. Accordingly, when the byte of 0x42 is separated, 0x04 and 0x02 are generated. As a result, the range of the maximum representable number of the divided data is reduced from 0x00 to 0xFF to 0x00 to 0x0F, and the representable number is reduced from a total of 256 to 16. Therefore, the entropy range of the generated ciphertext decreases from 0 to 8 to 0 to 4. However, since one input byte is output as two bytes in the process of dividing one byte, the size of the entire data can be doubled compared to the original data. Figure 4 shows entropy measurement results according to each file format using Byte Split.

To describe the experimental results in detail, blue indicates the entropy of plaintext, red indicates the entropy of the encrypted file, and green indicates the entropy of the file to which the Byte Split method is applied. Depending on the file format, the entropy of plaintext can be different from that of ciphertext. However, the entropy of ciphertext is increased for all file formats. Since the entropy of the file to which the Byte Split method is applied intentionally reduces the number expression range, the entropy appears closer to four. As a result, if the Byte Split method is applied to TXT and XLS file formats, which are file formats whose entropy of plaintext is close to four, the entropy approaches four, similar to the plaintext entropy. Thus, it cannot effectively detect ransomware-infected files.

#### 4.1.2. Binary to ASCII

**Figure 5:**
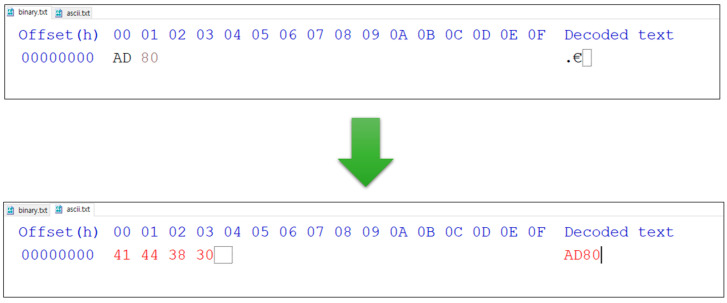
Input data conversion process of the BinaryToASCII technique.

The BinaryToASCII technique is a technique that manipulates entropy by reducing the expression range of a number, similar to the Byte Split technique. This technique does not divide bytes but replaces binary data with ASCII to reduce entropy by reducing the expression range of the number, which can be expressed by the existing one byte to the maximum possible one. In detail, the expression range of the number that is the result of the encrypted data of 8 bits (one byte) is 0x00 to 0xFF. If binary data are converted to ASCII, according to the ASCII code index table, numbers can be expressed from 0x30 to 0x39, and the alphabet can be expressed from 0x61 to 0x66 or from 0x41 to 0x46. In other words, a one-byte ciphertext can represent up to 256 characters. The BinaryToASCII technique can express numbers from “0” to “9”. The alphabet can be expressed from uppercase letters “A” to “F” or from the lowercase letters “a” to “f”. Accordingly, the range of representable numbers is reduced to a total of 16. To understand this process easier, the conversion process using HxD (Hex Editor) is shown in Figure 5.

**Figure 6:**
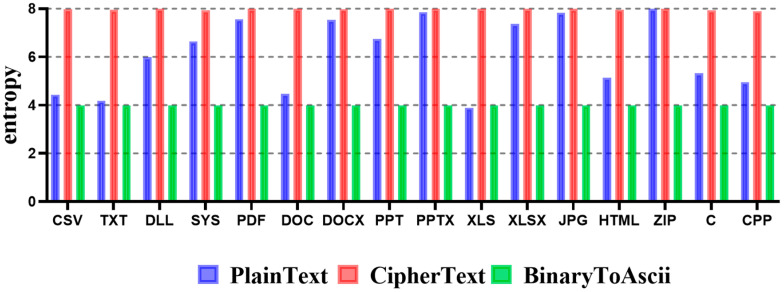
Entropy measurement results according to each file format using the BinaryToASCII technique.

This figure shows the results of outputting binary data with the BinaryToASCII technique applied to 0xAD80, which has 2 bytes that are assumed to be encrypted, with a hexadecimal editor. As a result of the conversion, “A” was converted to 0x41, “D” was 0x44, “8” was converted to 0x38, and “0” was converted to 0x30. The data actually stored would be 0x41443830. It can be confirmed that this belonged to 0x30 to 0x39, 0x61 to 0x66, or 0x41 to 0x46, which is the expression range of numbers due to the BinaryToASCII technique. However, this technique also has a feature where, similar to Byte Split, one byte inputted in the process of converting to ASCII is outputted as two bytes. Thus, the size of the ciphertext was doubled compared to the original data. Figure 6 shows the entropy measurement results for each file format using the BinaryToASCII technique.

Specifically, the entropy of the file to which the BinaryToASCII technique is applied intentionally reduced the number expression range. Thus, the entropy appeared close to four. This is because, similar to the Byte Split technique, the maximum expression range of numbers was reduced from 256 to 16. As a result, if the Byte Split technique is applied to TXT and XLS, which are file formats whose entropy of plaintext is close to four, the ciphertext entropy approaches four, similar to a plaintext entropy, thus effectively neutralizing the ransomware detection technology.

#### 4.1.3. Radix Conversion

**Figure 7:**
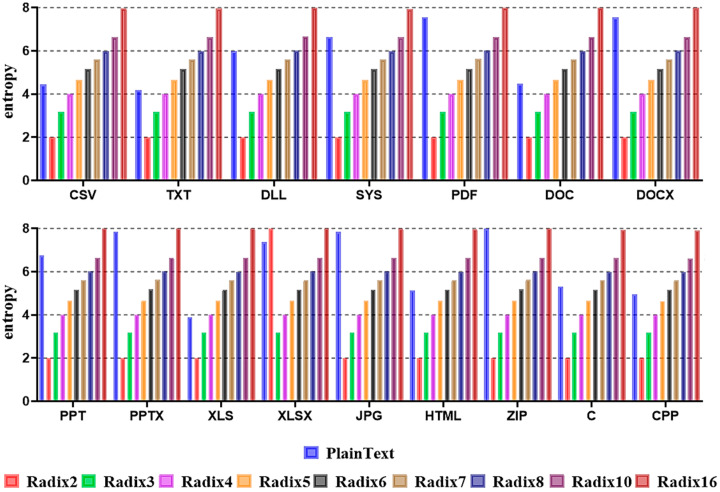
Entropy measurement results according to each file format using the Radix Conversion technique.

The third technique of the ransomware neutralization method based on format-preserving encryption is the Radix Conversion technique. As explained in Section 2.1.4, Format-Preserving Encryption, radix, means the number of domain codes. It has the same meaning as the radix notation in format-preserving encryption. The advantage of format-preserving encryption is that the radix for the encryption can be arbitrarily adjusted. For this reason, since the expression range of the input number can be arbitrarily adjusted, entropy also can be arbitrarily adjusted. In the end, we hypothesized that if the attacker adjusted the entropy of the ciphertext to be similar to the entropy of the plaintext, it could effectively neutralize the ransomware detection technology. Based on this fact, the entropy was measured while adjusting the radix to 2, 3, 4, 5, 6, 7, 8, 10, and 16 in order to select the best entropy for neutralization by the file format from the attacker’s point of view. Figure 7 shows the entropy measurement results according to the radix.

As a result of measuring entropy by adjusting the radix to 2, 3, 4, 5, 6, 7, 8, 10, and 16 for all file formats included in the dataset, the entropy increased and decreased according to the change in the radix, similar to that of plaintext, although there was a slight difference in the measured entropy. In other words, if the radix with an entropy similar to plaintext was selected based on the entropy, which varied according to the characteristics of each file format, it could effectively neutralize ransomware detection technology.

### 4.2. Derivation of the Optimal Neutralization Technique

Here, based on the proposed Byte Split, BinaryToASCII, and Radix Conversion techniques, a new method of neutralizing the ransomware detection technology using format-preserving encryption that could overcome the limitations of previous studies was verified based on the experiment results. Moreover, the optimal neutralization technique was derived by the comparison and analysis of each technique. The optimal neutralization technique to be derived should have the entropy most similar to the plaintext entropy, even if the ransomware creates an encrypted file using format-preserving encryption. Therefore, by measuring the entropy of each technique according to each file format, the technique most similar to the plaintext entropy could be selected as the optimal neutralization technique. Table 3 shows the entropy of files to which each technique can be applied, the plaintext entropy, and the difference. 

An analysis of the results showed that the Byte Split and BinaryToASCII techniques had exactly the same entropy for all the file formats included in the dataset. This result was because the expression range of the number that could be expressed in both techniques was the same as 16. Specifically, with Byte Split and BinaryToASCII techniques, the difference in entropy from the plaintext was relatively large except for the TXT file format.

With the Radix Conversion technique, plaintext and ciphertext entropy showed the most similar results when the CSV file format was set to radix 5, the DLL file format was set to radix 8, the SYS file format was set to radix 10, the PDF file format was set to radix 16, the DOC file format was set to radix 5, the DOX file format was set to radix 16, the PPT file format was set to radix 10, the PPTX file format was set to radix 16, the XLS file format was set to radix 4, the XLSX file format was set to radix 16, the HTML file format was set to radix 6, the C file format was set to radix 6, the CPP file format was set to radix 5, the JPG file format was set to radix 16, and the ZIP file format was set to radix 16.

**Figure 8:**
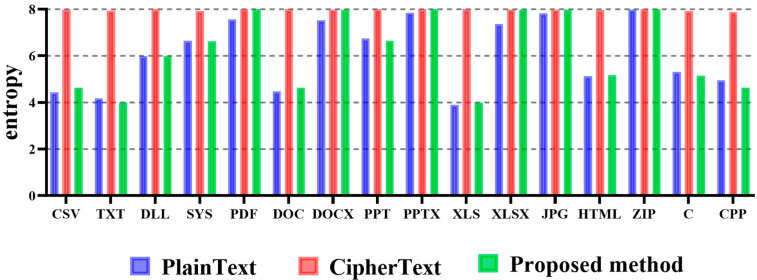
Entropy measurement results according to each file format using the proposed method.

To sum up the results, except for the TXT file format, the third method, the Radix Conversion technique, measured the optimal entropy for most file formats. Regarding this optimal technique in terms of file format, the optimal entropy was measured for six file formats with radix 16, radix 5 for each of the three file formats, radix 10 and radix 6 for each of the two file formats, and radix 8 and radix 4 for the one file format. Figure 8 shows the entropy measurement results for different files to which the derived optimal neutralization technique was applied by summarizing the experimental results.

In the above figure, we found that most file formats had entropy values that were almost similar to the entropy of the plaintext and the entropy of the optimal technique. The difference from the plaintext entropy was 0.01290 at the lowest and 0.63039 at the highest, which were not clearly distinguishable numbers considering that the plaintext entropy was the average entropy. As a result, we demonstrated through experiments that if an attacker who produced ransomware attempted a ransomware attack using the optimal neutralization technique proposed in this paper, the possibility of detection by the defense system for ransomware was minuscule.

## 5. Discussion

In this paper, to propose a new method with which to neutralize ransomware detection technologies, three techniques using format-preserving encryption were proposed, and the experimental results of each technique are analyzed in Section 4.1.1, Section 4.1.2 and Section 4.1.3. Based on the analysis results described in Section 4.2, the optimal neutralization technique was derived. This section describes the performance evaluation of each proposed neutralization technique. To evaluate the performance of each technique, the neutralization accuracy obtained in the previous study was compared to that obtained in the proposed technique. Here, the neutralization accuracy was calculated based on the difference between the plaintext entropy and the entropy to which each neutralization technique was applied so that a file infected by ransomware could be clearly detected based on the entropy threshold. For example, when the entropy threshold was 0.5, and the difference between the entropy of the plaintext and the entropy of the file to which the neutralization technique was applied was less than 0.5, the file was considered similar to the entropy of the plaintext. This meant that the defense system could not detect the file infected by the ransomware. It was, therefore, judged that the technique had been successfully neutralized.

**Figure 9:**
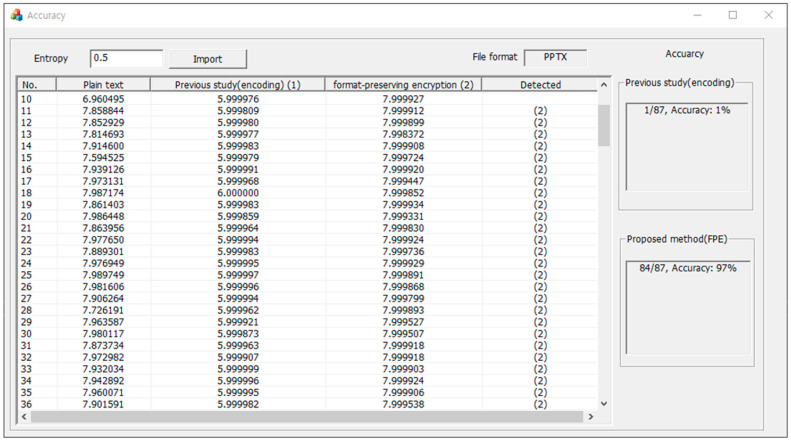
Example of comparison results of neutralization accuracy for the PPTX file format when the entropy threshold is set to 0.5.

In order to compare the neutralization accuracy with previous studies, a program was implemented to calculate the neutralization accuracy according to the defined entropy threshold. For a more accurate comparison, only the same files as those of the experimental dataset used in the previous study were experimented with. Figure 9 shows an example of the comparison result of the neutralization accuracy for the PPTX file format.

The entropy threshold is a reference value with which to measure the neutralization accuracy. A program detects a file infected with ransomware based on the difference from the plaintext entropy. However, since the measured entropy is different for each file format and even for the same file format, the neutralization accuracy might vary according to the defined entropy threshold. For this reason, considering the difference between the average entropy of plaintext and the average entropy of the neutralization technique, three entropy thresholds with optimal neutralization accuracy were set at 0.3, 0.4, and 0.5.

Figure 9 shows the evaluation results of the neutralization accuracy of the PPTX file format with the entropy threshold set at 0.5. The technique applying the encoding method described in a previous study [8] neutralized a total of 87 files, which showed a neutralization accuracy of about 1%. However, the proposed method using format-preserving encryption neutralized 84 files for a total of 87 files, which showed an accuracy of about 97%. Although only the PPTX file format was used as the subject, the proposed technique improved the neutralization accuracy by about 84 times compared to previous studies [8]. This meant that the proposed method was very effective from the point of view of neutralization. Based on more diverse file formats and thresholds, neutralization accuracy was measured. Table 4, Table 5 and Table 6 show the comparison results of the neutralization accuracy performance.

To summarize the results, we found that, among cases when the entropy threshold was defined as 0.3, 0.4, or 0.5, the case with an entropy threshold set at 0.5 had the highest neutralization accuracy. Specifically, the previous study [8] showed an accuracy of 31% for the CSV file format, while the proposed method showed an accuracy of 69%. For the DLL file format, both studies showed an accuracy of 36%. For the SYS file format, the accuracies of the previous study and the present study were 35% and 26%, respectively. For the PDF file format, the accuracy was 6% and 72%, respectively. For the DOC file format, the accuracy was 13% and 28%, respectively. For the DOCX file format, the accuracy was 0% and 67%, respectively. For the PPT file format, the accuracy was 8% and 21%, respectively. For the PPTX file format, the accuracy was 1% and 97%, respectively. For the XLS file format, the accuracy was 4% and 48%, respectively. For the XLSX file format, the accuracy was 0% and 57%, respectively. For the JPG file format, the accuracy was 0% and 91%, respectively. For the HTML file format, neutralization accuracies in the previous study and the present study were 6% and 92%, respectively. For the ZIP file format, the accuracy was 0% and 100%, respectively. For the C file format, the accuracy was 12% and 99%, respectively. Finally, for the CPP file format, the accuracy was 0% and 65%, respectively. Except for the DLL file format and the SYS file format, the present study showed the same performance as the previous study, and the accuracy of the proposed neutralization technique was further improved for all file formats compared to the previous study.

In this paper, based on the above experimental results, the limitations of previous studies were overcome, and the optimal ransomware detection-neutralization technique was derived. This is most similar to the entropy of plaintext in the file format by actively utilizing the advantages of format-preserving encryption. As a result, we demonstrated that the entropy measurement-based ransomware detection technology had very effective results from the point of view of neutralization.

## 6. Conclusions

This paper proposed a new method for neutralizing ransomware detection technologies that could overcome the limitations of previous studies by considering the attacker’s position as a ransomware producer. Previous studies have considered the attacker’s point of view. However, if entropy is measured after decoding the suspicion that a neutralization method was being applied in the system to defend the ransomware, there is a problem of neutralization failure because the encoding algorithm can be applied as a method for neutralization. Moreover, the DOCX, PDS, PPTX, XLSX, JPG, and ZIP file formats still exhibit relatively large differences between the entropy of the encoded file and the entropy of the plaintext. Thus, there is a problem when providing a clue to suspect that a neutralization method has been applied. To solve this problem, in this paper, the limitations of previous studies were actively analyzed. Based on the analyzed results, a new neutralization method using format-preserving encryption and three techniques were proposed. The applicability of the proposed technique was verified through experimental results, and the optimal neutralization technique was derived based on the experimental results. Moreover, as a result of comparing the performance with the previous study for performance evaluation, when the entropy threshold was set at 0.5, the proposed method showed a 96% improvement in neutralization accuracy compared to the previous method based on the PPTX file format. The results of this study could ultimately lead to the development of new technologies for detecting and threatening files infected by ransomware.

In the future, we plan to study the detection methods of files infected with ransomware or those infected with ransomware by applying machine learning models based on the results of the method proposed in this paper to counteract the neutralization technology of entropy-based ransomware detection.

## Figures and Tables

**Table 1:** Dataset used in experiments.

File Type	File Format	Number of Files
Text file	csv	800
	txt	800
System file	sys	800
	dll	450
Document file	pdf	450
	doc	450
	docx	150
	ppt	450
	pptx	150
	xls	150
	xlsx	30
Image file	jpg	450
Webpage file	html	800
Compressed file	zip	5
Source code file	c	150
	cpp	150

**Table 2:** Proposed three new techniques for manipulating entropy.

Technique		Number of Representable Numbers	Range of Representable Numbers	Change in Ciphertext Size	Description
Byte Split		16	0x00~0x0 F	×2	Split each half byte (4 bits) and replace the first 4 bits with 0 Example) 42 71 (16) → 04 02 07 01 (16) Representable range: 256 → 16 Change in ciphertext size: 2×
BinaryToASCII		16	0x30~0x39 (Decimal) 0x61~0x66 (Alphabet)	×2	After encryption, the ciphertext is converted to an ASCII code and stored Binary to ASCII() function Representable range: 256 → 16 Change in ciphertext size: 2×
Radix Conversion	Radix2	2	0x00~0x01	×3.56	Utilize the advantages of format-preserving encryption that can change the radix Diversification of expressive range due to free radix change A different entropy is generated according to radix Change in ciphertext size: different for each radix based on random 16 bytes of plaintext
	Radix3	3	0x00~0x02	×2.25	
	Radix4	4	0x00~0x03	×1.81	
	Radix5	5	0x00~0x04	×1.56	
	Radix6	6	0x00~0x05	×1.37	
	Radix7	7	0x00~0x06	×1.25	
	Radix8	8	0x00~0x07	×1.18	
	Radix10	10	0x00~0x09	×1.06	
	Radix16	16	0x00~0x0F	×1	

**Table 3:** Comparison of entropy measurement results of previous studies and each technique applying format-preserving encryption.

File Format	Plain Text	Byte Split	Binary to Ascii	Radix Conversion								
				2	3	4	5	6	7	8	10	16
CSV	4.43359		3.99924 (+0.43434)	1.99992 (+2.43366)	3.16960 (+1.26399)	3.99923 (+0.43435)	4.64247 **(−0.20888)**	5.16760 (−0.73401)	5.61130 (−1.17772)	5.99520 (−1.56162)	6.63543 (−2.20184)	7.97352 (−3.53993)
TXT	4.18490		3.99846 **(+0.18643)**	1.99985 (+1.01573)	3.16917 (+1.01573)	3.99834 (+0.18656)	4.64099 (−0.45609)	5.16556 (−0.98066)	5.60713 (−1.42224)	5.99043 (−1.80553)	6.62661 (−2.44171)	7.94958 (−3.76468)
DLL	5.98047		3.99986 (+1.98062)	1.99997 (+2.81066)	3.16981 (+2.81066)	3.99978 (+1.98069)	4.64343 (+1.33704)	5.16936 (+0.81111)	5.61394 (+0.36653)	5.99884 **(−0.01836)**	6.67259 (−0.69212)	7.99505 (−2.01458)
SYS	6.64239		3.99814 (+2.64425)	1.99982 (+3.47322)	3.16918 (+3.47322)	3.99804 (+2.64435)	4.64005 (+2.00235)	5.16379 (+1.47861)	5.60583 (+1.03656)	5.98736 (+0.65503)	6.62082 **(+0.02157)**	7.92800 (−1.28561)
PDF	7.56255		3.99992 (+3.56264)	1.99997 (+4.39272)	3.16983 (+4.39272)	3.99983 (+3.56272)	4.64354 (+2.91901)	5.16947 (+2.39308)	5.61408 (+1.94847)	5.99914 (+1.56342)	6.64250 (+0.92005)	7.99701 **(−0.43445)**
DOC	4.47150		3.99992 (+0.47158)	1.99981 (+1.30254)	3.16897 (+1.30254)	3.99832 (+0.47318)	4.64133 **(−0.16983)**	5.16727 (−0.69577)	5.61165 (−1.14015)	5.99561 (−1.52411)	6.63978 (−2.16828)	7.99721 (−3.52570)
DOCX	7.53530		3.99942 (+3.53588)	1.99993 (+4.36558)	3.16972 (+4.36558)	3.99959 (+3.53571)	4.64286 (+2.89245)	5.16858 (+2.36673)	5.61261 (+1.92269)	5.99702 (+1.53829)	6.63872 (+0.89658)	7.98427 **(−0.44897)**
PPT	6.74016		3.99998 (+2.74018)	1.99997 (+3.57032)	3.16984 (+3.57032)	3.99989 (+2.74027)	4.64365 (+2.09651)	5.16858 (+1.57159)	5.61261 (+1.12755)	5.99959 (+0.74057)	6.64333 **(+0.09683)**	7.99927 (−1.12911)
PPTX	7.85963		3.99998 (+3.85965)	1.99999 (+4.68972)	3.16991 (+4.68972)	3.99997 (+3.85966)	4.64379 (+3.21584)	5.16984 (+2.68979)	5.61461 (+2.24502)	5.99986 (+1.85977)	6.64365 (+1.21599)	7.99945 **(−0.13981)**
XLS	3.89697		3.99987 (−0.10290)	1.99977 (+0.72828)	3.16869 (+0.72828)	3.99770 **(−0.10072)**	4.64041 (−0.74343)	5.16610 (−1.26913)	5.61040 (−1.71343)	5.99346 (−2.09649)	6.63766 (−2.74069)	7.99521 (−4.09824)
XLSX	7.36146		3.99974 (+3.36175)	7.99184 (+4.19169)	3.16977 (+4.19169)	3.99970 (+3.36176)	4.64331 (+2.71815)	5.16921 (+2.19225)	5.61361 (+1.74785)	5.99844 (+1.36302)	6.64132 (+0.72014)	7.99184 **(−0.63039)**
JPG	7.83111		3.99965 (+3.38146)	1.99996 (+4.66133)	3.16978 (+4.66133)	3.99964 (+3.83147)	4.64318 (+3.18792)	5.16883 (+2.66228)	5.61321 (+2.21790)	5.99780 (+1.83331)	6.64006 (+1.19105)	7.98816 **(−0.15705)**
HTML	5.13085		3.99907 (+1.13178)	1.99990 (+1.96132)	3.16952 (+1.96132)	3.99902 (+1.13182)	4.64218 (+0.48867)	5.16675 **(−0.03590)**	5.61022 (−0.47937)	5.99357 (−0.86273)	6.63271 (−1.50187)	7.96442 (−2.83358)
ZIP	7.98678		3.99998 (+3.98679)	2.00000 (+4.81686)	3.16992 (+4.81686)	3.99999 (+3.98679)	4.64382 (+3.34295)	5.16988 (+2.81690)	5.61465 (+2.37212)	5.99992 (+1.98685)	6.64375 (+1.34302)	7.99967 **(−0.01290)**
C	5.31892		3.99806 (+1.32086)	1.99977 (+2.14983)	3.16909 (+2.14983)	3.99787 (+1.32106)	4.64029 (+0.67863)	5.16410 **(+0.15483)**	5.60583 (−0.28691)	5.98767 (−0.66875)	6.62328 (−1.30436)	7.93207 (−2.61315)
CPP	4.94971		3.99615 (+0.95356)	1.99969 (+1.78154)	3.16816 (+1.78154)	3.99459 (+0.95512)	4.63443 **(+0.31528)**	5.15712 (−0.20741)	5.59549 (−0.64578)	5.97598 (−1.02627)	6.60374 (−1.65403)	7.89892 (−2.94921)

The highlighted part in blue means the highest performance encoding method.

**Table 4:** Comparison of ransomware detection-neutralization accuracy with previous studies (entropy threshold: 0.3).

Entropy Threshold	File Type	File Format	Previous Study (Best Encoding Method)	Proposed Method (Format-Preserving Encryption)
0.3	Text file	CSV	13%	**49%**
		TXT	0%	**16%**
	System file	DLL	30%	
		SYS	**25%**	13%
	Document file	PDF	3%	**53%**
		DOC	6%	**23%**
		DOCX	0%	**47%**
		PPT	3%	**14%**
		PPTX	0%	**85%**
		XLS	1%	**25%**
		XLSX	0%	**43%**
	Image file	JPG	0%	**90%**
	Web page file	HTML	0%	**76%**
	Compressed file	ZIP	0%	**100%**
	Source code file	C	0%	**63%**
		CPP	0%	**33%**

The blue highlighted part means a relatively high-performance method.

**Table 5:** Comparison of ransomware detection-neutralization accuracy with previous studies (entropy threshold: 0.4).

Entropy Threshold	File Type	File Format	Previous Study (Best Encoding Method)	Proposed Method (Format-Preserving Encryption)
0.4	Text file	CSV	18%	**63%**
		TXT	0%	**31%**
	System file	DLL	32%	
		SYS	**30%**	21%
	Document file	PDF	3%	**63%**
		DOC	10%	**25%**
		DOCX	0%	**58%**
		PPT	6%	**17%**
		PPTX	0%	**93%**
		XLS	2%	**37%**
		XLSX	0%	**48%**
	Image file	JPG	0%	**91%**
	Web page file	HTML	2%	**86%**
	Compressed file	ZIP	0%	**100%**
	Source code file	C	0%	**99%**
		CPP	0%	**43%**

The blue highlighted part means a relatively high-performance method.

**Table 6:** Comparison of ransomware detection-neutralization accuracy with previous studies (entropy threshold: 0.5).

Entropy Threshold	File Type	File Format	Previous Study (Best Encoding Method)	Proposed Method (Format-Preserving Encryption)
0.5	Text file	CSV	31%	**69%**
		TXT	0%	**48%**
	System file	DLL	36%	
		SYS	**35%**	26%
	Document file	PDF	6%	**72%**
		DOC	13%	**28%**
		DOCX	0%	**67%**
		PPT	8%	**21%**
		PPTX	1%	**97%**
		XLS	4%	**48%**
		XLSX	0%	**57%**
	Image file	JPG	0%	**91%**
	Web page file	HTML	6%	**92%**
	Compressed file	ZIP	0%	**100%**
	Source code file	C	12%	**99%**
		CPP	0%	**65%**

The blue highlighted part means a relatively high-performance method.

## Data Availability

Data are contained within the article.
